# Experimental, Theoretical, and *In Silico* Studies of Potential CDC7 Kinase Inhibitors

**DOI:** 10.1021/acsomega.4c07221

**Published:** 2024-12-31

**Authors:** Kannika Byadarahalli Ravindranath, Saravanan Kandasamy, Hossam Ebaid, Jameel Al-Tamimi, Sanjeev Murthy Talya Narasimhamurthy, Manju Nagaraja, Ahmad Hosseinizadeh, Madan Kumar Shankar

**Affiliations:** † Department of Pharmaceutical Biosciences, Uppsala University, Husargatan 3, Uppsala 752 37, Sweden; ‡ Biological and Chemical Research Center, Faculty of Chemistry, University of Warsaw, Warsaw 02-089, Poland; § Zoology Department, College of Science, King Saud University, Riyadh 2455, Saudi Arabia; ∥ Department of Chemistry, Sri Siddhartha Academy of Higher education, Tumkur 572107, India; ⊥ Department of Chemistry, Govt. Degree College, Lingasugur, Karnataka 584122, India; # Department of Physics, University of Wisconsin-Milwaukee, Milwaukee, Wisconsin 53211, United States; ∇ Department of Chemistry-BMC Biochemistry, University of Uppsala, Husargatan 3, Uppsala 75237, Sweden

## Abstract

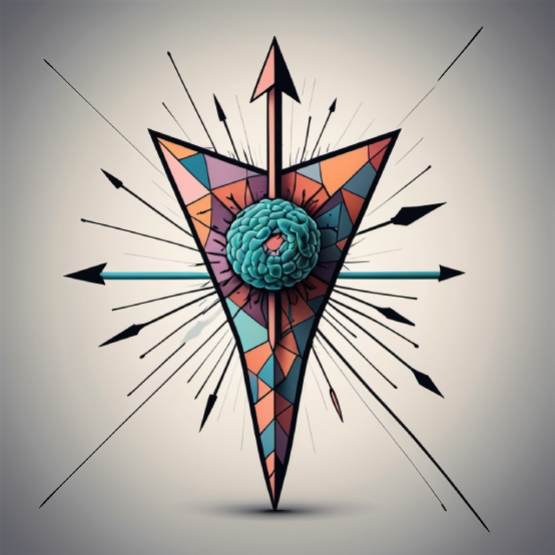

In this work, we present the synthesis, solid-state characterization, and *in silico* studies of two pyrazole derivatives: 5-(2-methylphenoxy)-3-methyl-1-phenyl-1*H*-pyrazole-4-carbaldehyde (I) and 5-(4-methylphenoxy)-3-methyl-1-phenyl-1*H*-pyrazole-4-carbaldehyde (II). The molecular crystal properties, in terms of intermolecular hydrogen bonds and other weak interactions, are analyzed using single crystal X-ray diffraction. The Hirshfeld surfaces computational method is used to quantify the intermolecular interactions, density functional theory for theoretical structural optimization, and its comparison with the experimental structure and *in-silico* studies using docking and molecular dynamics studies of I and II with CDC7-kinase. In addition, the quantum theory of atoms in molecules (QTAIM) approach is applied to calculate the topological properties of electron density and the Laplacian of electron density of the chemical bonds of both molecules. Compounds I and II crystallize in a monoclinic crystal system, and molecules are connected via C–H···O intermolecular hydrogen bonds. Hirshfeld surfaces analysis revealed that the H···H type intercontact contributes more toward the crystal packing. DFT-optimized structures show a perfect overlay with the experimental structures. The *in silico* results show that both I (−41.50 kcal/mol) and II (−44.53 kcal/mol) exhibit strong binding free energies as ligands binding to the active sites of the CDC7-kinase. The most significant contributions for ligand and protein binding in both compounds are dominated by van der Waals interactions.

## 1 Introduction

Pyrazole derivatives have been used as a core for synthesizing various other derivatives. These compounds exhibit various pharmacological activities: anti-inflammatory,^1^ antibacterial,^2^ analgesic,^3^ anticonvulsant,^4^ anticancer,^5−10^ and antidepressant activities.^11^ The biological and chemical properties of these compounds are often controlled by how they are altered. For e.g., the substitution of an aryl group in the pyrazole ring’s fourth position increases its inhibition of LDH by 100-fold.^12^ However, changes at the first or third position abolish the same.^13^ Intermolecular steric interactions play major roles in the specific properties of these molecules. Therefore, exploring these intra- and intermolecular interactions will be worthwhile for the scientific community to design future drugs. Also, there is no simpler route to synthesize pyrazole derivatives, which is still a major concern.^14,15^ One of the common routes for synthesizing these molecules is the reaction of 1,3-diketones with hydrazine derivatives.^16−18^ The green synthesis of the pyrazole derivatives is of much interest,^19^ and a few of these routes are ultrasound and microwave synthesis.^20,21^

CDC7 kinase, a serine/threonine protein kinase, is essential for the regulation of the cell cycle, particularly in the initiation of DNA replication. As a member of the cyclin-dependent kinase (CDK) family, CDC7 is pivotal in phosphorylating the minichromosome maintenance (MCM) complex, which is vital for DNA unwinding and the initiation of DNA synthesis during the S phase of the cell cycle.^22^ This mechanism is crucial for preserving genomic stability and facilitating proper cell division. The activity of CDC7 is meticulously controlled through various mechanisms, including its interaction with specific cyclins and other regulatory proteins. Typically, its expression and activity increase in response to growth signals that encourage cell cycle progression.^23^ The first effective CDC7 inhibitors, known as 2-heteroaryl-pyrrolopyridinones, were introduced in 2008.^24^ Small molecule inhibitors are designed to interfere with CDC7’s activity, thereby obstructing the phosphorylation of the MCM complex and hindering DNA replication. Such interventions can result in cell cycle arrest and, ultimately, apoptosis in cancer cells. Several CDC7 inhibitors have been developed and evaluated in preclinical studies, showing encouraging efficacy across various cancer models, including those resistant to standard therapies. The report on pyrrolopyridinones and 5-heteroaryl-3-carboxamido-2-aryl pyrroles, which have demonstrated significant inhibitory effects on CDC7 and exhibit antitumor properties both *in vitro* and *in vivo*.^25,26^ Nonetheless, the cellular response to the inhibition of CDC7 is intricate, as DNA replication and cell proliferation can still occur despite diminished CDC7 activity.^27^ The phosphorylation of MCM2, which serves as a biomarker for CDC7 activity, is highly responsive to inhibition; however, it may not provide an accurate representation of the effects on DNA synthesis.^27^ Additionally, thiazole-based compounds have been investigated as potential CDC7 inhibitors, with some exhibiting strong and selective inhibition of CDC7 kinase activity, reduced MCM2 phosphorylation, and a decrease in both DNA synthesis and cell viability.^28^ A significant category of CDC7 inhibitors comprises ATP-competitive small molecules that imitate the natural substrate of the kinase, thereby effectively inhibiting its catalytic function. Ongoing research into CDC7 inhibitors is advancing, with a focus on elucidating the molecular mechanisms that drive their effects and identifying biomarkers that may predict patient responses. The therapeutic potential is substantial, as targeting CDC7 could enable the selective destruction of cancer cells while preserving normal tissues, ultimately improving treatment outcomes and reducing side effects.

The capability of computational techniques to explore diverse molecular properties is of considerable significance. Notably, research that integrates both experimental and computational approaches holds particular importance. There are similar kinds of studies performed for different kinds of analogs.^29−31^ Our study presents the synthesis and experimental characterization, including crystal structure, *in silico* (docking and MD simulations), and theoretical studies (DFT and QTAIM) of 5-(2-methylphenoxy)-3-methyl-1-phenyl-1*H*-pyrazole-4-carbaldehyde and 5-(4-methylphenoxy)-3-methyl-1-phenyl-1*H*-pyrazole-4-carbaldehyde.

## 2 Experimental Section

### 2.1 Synthesis, Crystallization, and Spectroscopy

#### 2.1.1 Synthesis and Crystallization of I and II

**Scheme 1:**
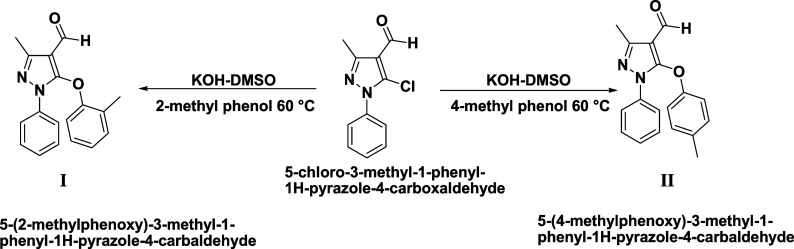
Synthetic Route of I and II

The solution of 2/4-methylphenol (0.0025 mol) was dissolved in 10 mL of dimethyl sulfoxide; 5-chloro-3-methyl-1-phenyl-1*H*-pyrazol-4-carbaldehyde (0.002 mol) and potassium hydroxide (0.002 mol) were added and heated on an oil bath at 60 °C for 6 h (Scheme 1). The final resulting reaction mixture was cooled to room temperature. Later, the reaction mixture was poured into crushed ice, and the separated solid was filtered and washed with water. The dried product was recrystallized from ethanol.

#### 2.1.2 Spectroscopy Data of Compound (I): 5-(2-Methylphenoxy)-3-methyl-1-phenyl-1*H*-pyrazole-4-carbaldehyde

^1^H NMR: 400 MHz, CDCl_3_ δ ppm: 2.32 (s, 3H, *p*-tolyl), 2.56 (s, 3H, CH_3_), 6.94–6.96 (d, 2H, *J* = 6.80 Hz, *ortho* protons of *p*-tolyl), 7.13–7.14 (d, 2H, *J* = 6.80 Hz, *meta* protons of *p*-tolyl), 7.35–7.36 (d, 1H, *J* = 6.00 Hz), 7.42–7.45 (t, 2H, *meta* protons of *N*-phenyl), 7.65–7.67 (d, 2H, *J* = 6.40 Hz, *ortho* protons of N-phenyl), 9.56 (s, 1H, aldehyde) (Figure S9).

^13^C NMR: 100 MHz, CDCl_3_ δ ppm: 14.65 (pyrazole methyl), 20.63 (*p*-tolyl), 108.71, 116.17, 122.79, 127.94, 129.23, 130.70, 134.43, 136.98, 150.81, 152.99, 155.03, 183.25 (aldehyde carbon) (Figure S10).

Mass (*m*/*z*) 293 (M^+^ + 1) (M.F. C_18_H_16_N_2_O_2_), Yield: 74%, M.P.: 52 °C.

#### 2.1.3 Spectroscopy Data of Compound (II): 5-(4-Methylphenoxy)-3-methyl-1-phenyl-1*H*-pyrazole-4-carbaldehyde

^1^H NMR: 400 MHz, CDCl_3_ δ ppm: 2.37 (s, 3H, *p*-tolyl), 2.56 (s, 3H, CH_3_), 6.84–6.85 (d, 1H, *J* = 6.40 Hz, *o*-tolyl proton), 7.06–7.09 (m, 1H, *o*-tolyl proton), 7.12–7.15 (m, 1H, *o*-tolyl proton), 7.25–7.28 (m, 1H, *o*-tolyl proton), 7.34–7.37 (m, 1H, H-4 of *N*-phenyl), 7.43–7.46 (t, 2H, *meta* protons of *N*-phenyl), 7.66–7.67 (d, 2H, *J* = 6.40 Hz, *ortho* protons of *N*-phenyl), 9.43 (s, 1H, aldehyde) (Figure S11).

^13^C NMR: 100 MHz, CDCl_3_ δ ppm: 14.71 (pyrazole methyl), 16.04 (*p*-tolyl), 108.48, 115.34, 122.85, 124.91, 127.35, 127.61, 127.97, 129.22, 131.93, 137.02, 150.85, 153.12, 155.30, 183.07 (aldehyde carbon) (Figure S12).

Mass (*m*/*z*) 293 (M^+^ + 1) (M.F. C_18_H_16_N_2_O_2_), Yield: 81%, M.P.: 64 °C.

### 2.2 X-ray Intensity Data Collection

#### 2.2.1 Data Collection, Structure Solution, and Refinement

The diffracted X-ray intensities for the carefully selected crystal with dimensions 0.32 × 0.24 × 0.23 mm^3^ (I) and 0.31 × 0.23 × 0.21 mm^3^ (II) were collected using a Rigaku diffractometer (Saturn 724+ CCD detector) at 293 K (Table 1). In I, a total of 20674 reflections were collected with a θ range of 2.8° to 31.1°, out of which 4550 were found to be unique, and using the *I* > 2s (*I*) criterion, 2735 reflections were treated as observed. A total of 14952 reflections were collected with a θ range of 2.3° to 25.7°, out of which 2856 were found to be unique, and using the *I* > 2σ (*I*) criterion, 1844 reflections were treated as observed in II.

**Table 1:** Crystal Data and Structure Determination and Refinement Details of I and II

Crystal Data		
CCDC	1519526	1519206
Empirical formula	C_18_H_16_N_2_O_2_	C_18_H_16_N_2_O_2_
Formula weight	292.33	292.33
Temperature K	293(2)	293
Crystal system	Monoclinic	Monoclinic
Space group	*P*2_1_/*n*	*P*2_1_/*c*
**Unit Cell Dimensions**		
a Å	9.7655(8)	9.1758(4)
b Å	13.5286(12)	7.6105(4)
c Å	11.6107(10)	22.0250(12)
α°	90	90
β°	97.792(9)	94.506(5)
γ°	90	90
Volume Å^3^	1519.8(2)	1533.31(13)
Z (No. of molecules in unit cell)	4	4
Density (ρ_calc_) g/cm^3^	1.278	1.266
μmm^–1^ (absorption coefficient)	0.085	0.084
F(000)	616	616
Crystal size (mm^3^)	0.31 × 0.23 × 0.21	0.32 × 0.24 × 0.23
Morphology and Color	Block and colorless	Block and colorless
**Data Collection and Refinement**		
Diffractometer	Rigaku Saturn 724^+^	Rigaku Saturn 724^+^
Radiation Source/ Monochromator	Sealed tube/Graphite	Sealed tube/Graphite
Adsorption correction (*SADABS*; Bruker, 2012)	Multiscan	Multiscan
*T*_min_ and *T*_max_	0.977 and 0.982	0.976 and 0.981
θ_min_ and θ_max_ (deg)	2.3 and 25.7	2.8 and 31.1
Radiation	MoKα (λ = 0.71073)	MoKα (λ = 0.71073)
*h*_min_ ≤ *h* ≤ *h*_max_	–11 ≤ *h* ≤ 11	–12 ≤ *h* ≤ 13
*k*_min_ ≤ *k* ≤ *k*_max_	–16 ≤ *k* ≤ 16	–8 ≤ *k* ≤ 10
*l*_min_ ≤ *l* ≤ *l*_max_	–14 ≤ *l* ≤ 14	–31 ≤ *l* ≤ 31
Measured reflections	14952	20674
Independent reflections [R_int_]	2856 [0.129]	4550 [0.049]
Restraints/parameters	0/212	0/201
Refinement method	F^2^	F^2^
Goodness-of-fit on F^2^	1.09	1.05
Final R indexes [I ≥ 2σ (I)]	R_1_ = 0.0791, wR_2_ = 0.2679	R_1_ = 0.0575, wR_2_ = 0.1569
Largest diff. peak/hole e Å^–3^	0.29 /–0.36	0.18 /–0.20

I and II: The final reflection file was the input to the SHELXS^32^ program to solve the crystal structure. The electron density peaks were labeled as expected and refined isotropically using the program SHELXL^33^ by full-matrix least-squares refinement against F^2^. Further, all the non-hydrogen atoms were refined anisotropically, and the chemically acceptable positions for hydrogen atoms were assigned. [The distances of C–H lie within the range 0.93–0.96 Å; *Uiso* (H) = 1.5 *Ueq* (C) (for H atoms) values were applied to ride on their parent atoms]. The PLATON^34^ program was used to calculate molecular geometry. The molecular diagram [ORTEP: thermal ellipsoid plots] and molecular packing diagrams were generated using MERCURY.^35^ Intermolecular hydrogen bonds are listed in Table 2. In compound II, all the non-hydrogen atoms were refined anisotropically, and the chemically acceptable positions for hydrogen atoms were assigned [The distances of C–H lie within the range 0.93–0.96 Å; *Uiso* (H) = 1.2 *Ueq* (C) values were applied to ride on their parent atoms]. The disorder atoms were refined, and the occupancies were: O2A (0.432(10)) and O2B (0.568(10)).

**Table 2:** Intermolecular Hydrogen Bonding Interaction Parameters of I and II

D	H	A	d(D-H)/Å	d(H-A)/Å	d(D-A)/Å	D-H-A/°
C8–	H8···	O2B^a^	0.93	2.50	3.163 (6)	129
C13–	H13···	O1^a^	0.93	2.61	3.533 (1)	168.35

a 3 *x*, −1 + *y*, *z*.

### 2.3 Hirshfeld Surfaces Analysis

In the crystal structure, intermolecular interactions are resolved into interatomic contacts using the Hirshfeld surfaces computational method.^36^ This method is used to visualize and quantify the interatomic contacts in terms of Hirshfeld surfaces and 2D fingerprint plots.^37^ The region over the Hirshfeld surfaces is marked with red and blue colors. The atom-to-atom contacts (interatomic contacts) in the crystal structure are plotted using two-dimensional (2D) fingerprint plots. The interatomic contacts are quantified in terms of percentage contribution toward the Hirshfeld surfaces. The parameters defined to plot 2D fingerprint plots are distances from the Hirshfeld surface to the nearest nucleus inside (*di*) and distances from the Hirshfeld surface to the nearest nucleus outside the surfaces (*de*). These parameters are used to calculate the percentage contribution values. The Hirshfeld surface signifies the intermolecular contacts of the molecules, and it is represented using conventional *dnorm* mapping. Crystal Explorer is used to calculate Hirshfeld surfaces and their fingerprint plots using the final crystallographic information file (CIF) as input.^38^

### 2.4 QTAIM and NCI Analysis

The DFT calculations for the crystallographic coordinates of both compounds were carried out using the Gaussian16 program^39^ (PLGrid portal, Ares supercomputer) at the B3LYP/6–311++G(d,p) level of theory.^40−43^ The HOMO and LUMO maps were plotted by Gauss View 5.0.^44^ The molecular electrostatic potential was computed at the same level of theory and plotted using a 3D plot.^45^ Furthermore, alongside the DFT, the QTAIM and NCI plot isosurfaces have been employed to analyze noncovalent interactions at the identical theoretical level. The NCI plot isosurfaces offer insights into both advantageous and disadvantageous interactions, distinguished by the sign of the second-density Hessian eigenvalue and characterized by the color of the isosurface.^46^ The color gradient employed follows a red-yellow-green-blue scale, where red indicates positive electron density (repulsive) and blue represents negative electron density (attractive). In the protein–ligand complex, the MD trajectory was subjected to clustering, and the average PDB coordinates were utilized for QTAIM analysis.

### 2.5 Docking and MD Simulation

Molecular docking is a method used in rational drug design and mechanistic analysis. It involves placing a target ligand molecule into the active site of a protein or DNA in a noncovalent manner. In this study, pyrazole derivatives were selected for docking analysis. These ligands were optimized and minimized using the *LigPrep* program (Schrödinger Release 2023-1: LigPrep, Schrödinger, LLC, New York, NY) and the OPLS4 force field. The crystal structure of CDC7 kinase (PDB: 355D) was obtained from the Protein Data Bank.^47^ Formal bond orders were assigned, water molecules beyond 5 Å from the het groups were deleted, and hydrogen atoms, charges, and missing side chains of the residues were added. The prepared structures were then optimized and minimized using the OPLS4 force field and the protein preparation wizard of the Schrödinger Suite Release 2023-1.^48^ The prepared ligands and CDC7 kinase were used for molecular docking studies, specifically employing the induced fit docking (IFD) method. IFD is known for its accuracy in predicting the binding mode of targets. The standard protocol was followed with a grid box centered around the binding site. The van der Waals scaling for both CDC7 kinase and ligands was set at 0.50. The docking was performed in the extra precision (XP) mode. The intermolecular interactions of the CDC7-ligand complexes were analyzed using protein–ligand interaction profiler (PLIP), Discovery Studio Visualizer, and PyMOL software.^49−52^

The stability of docked protein–ligand complexes was assessed by subjecting the synthesized pyrazole derivatives to molecular dynamics (MD) simulation using Amber22.^53^ To create the simulation environment, the protein–ligand complexes were placed in a cubic box with 2.5 Å solvent buffers using the TIP3P water model. The system was then neutralized by adding Na^+^ and Cl^–^ ions at a concentration of 0.15 M. To ensure relaxation of the solvated system, a six-step procedure implemented by Desmond was employed. This involved subjecting the protein–ligand complexes to 1000 steepest descent energy minimization steps, with a maximum force threshold of 100 kJ/mol·nm, before thermalization. Following this, the system was simulated in the isothermal–isobaric (NPT) ensemble at a constant temperature of 300 K and pressure of 1 bar using a time step of 2 fs. Subsequently, a simulation of the canonical ensemble NVT (moles, volume, and temperature) was performed for 1 ns. Finally, the production phase of the simulation was carried out for 100 ns to ensure equilibration of the system.^54^ Additionally, the MM-GBSA method was utilized to determine the binding free energies of both synthesized compounds. This technique presents several advantages compared to alternative methods, including reduced time requirements and lower computational costs, which enhance its applicability. For each complex, a complete simulation trajectory was employed to assess the van der Waals and electrostatic contributions.

## 3 Results and Discussion

### 3.1 Structure Details and Analysis

**Figure 1:**
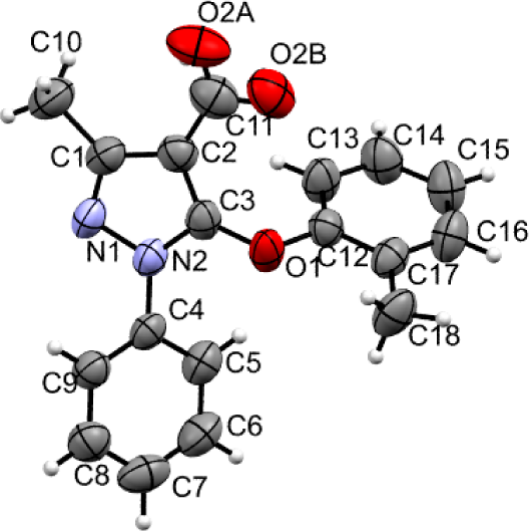
Atoms of molecule I represented with thermal ellipsoid plot (50% probability). The disorder atom O2A and O2B are shown, and their modeled disordered hydrogen atoms are not shown for the sake of clarity.

I: The thermal ellipsoid plot [*ORTEP*] of I was plotted as shown in Figure 1. The substructure of this molecule is labeled in terms of Cg1: pyrazole ring (N1–N2/C1–C3) and two phenyl rings (Cg2: C4–C9 and Cg3: C12–C17). Cg1 made dihedral angles of 45.95(14)° and 70.97(15)° with Cg2 and Cg3, respectively. The dihedral angle between Cg2 and Cg3 was 58.85(15)°. The Cg1 moiety (pyrazole ring) was planar with a maximum deviation of 0.004(2) Å for atom N1 from the mean plane. The rms deviation value of 0.002 Å was measured for a mean plane fitted through all non-hydrogen atoms of the Cg1. The Cg2 and Cg3 were essentially planar with maximum deviations of −0.014(4) Å and 0.009(3) Å for atoms C7 and C12, respectively [mean plane fitted through all non-hydrogen atoms; r.m.s. deviations were 0.003 Å (Cg2) and 0.003 Å (Cg3)]. The C–C bond lengths of Cg1 ranged from 1.370(3) Å to 1.384(2) Å. The bond angles of the Cg1 lay between 118.81(15)° and 121.03(14)° (average bond angle = 120°). Similarly, C–C bond lengths of Cg2 ranged from 1.367(2) to 121.50(16) Å. The bond angles of the Cg2 lay between 117.82(15)° and 121.50(16)° (average bond angle = 120°).

**Figure 2:**
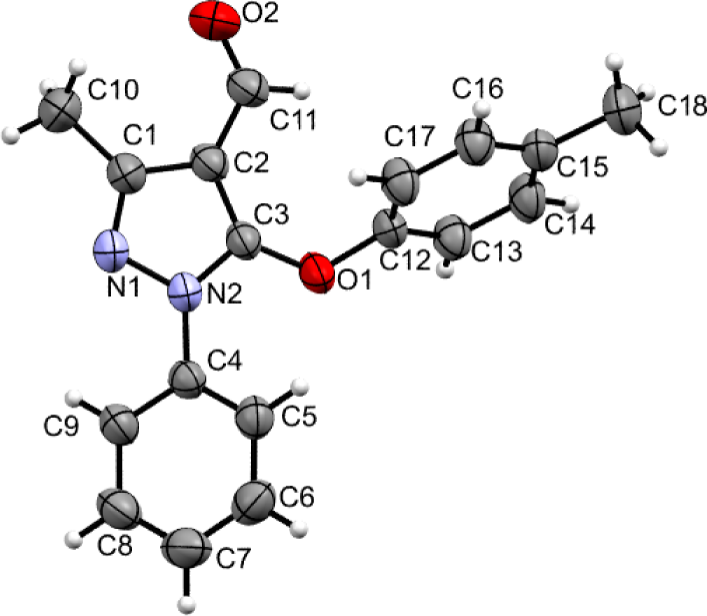
Atoms of molecule II represented with thermal ellipsoid plot (50% probability).

II: The thermal ellipsoid plot [*ORTEP*] of II was plotted as shown in Figure 2. It consisted of a pyrazole ring (Cg1: N1–N2/C1-C3) and two phenyl rings (Cg2: C4–C9 and Cg3: C12–C17). The Cg1 made dihedral angles of 41.31(8)° and 65.55(8)° with Cg2 and Cg3, respectively. The dihedral angle between Cg2 and Cg3 was 76.59(8)°. The Cg1 moiety (pyrazole ring) was planar with a maximum deviation of 0.009(1) Å for atom N1 from the mean plane. The rms deviation value of 0.001 Å was measured for a mean plane fitted through all non-hydrogen atoms of the Cg1. The Cg2 and Cg3 were essentially planar with maximum deviations of −0.008(2) and −0.008(3) Å for atoms C7 and C16, respectively [mean plane fitted through all non-hydrogen atoms rms deviations were 0.002 Å (Cg2) and 0.002 Å (Cg3)]. The C–C bond lengths of Cg1 ranged in the limits of 1.370(2) Å to 1.384(2) Å. The bond angles of the Cg1 lay between 118.82(15)° and 121.04(14)° (average bond angle = 120°). Similarly, C–C bond lengths of Cg2 ranged in the limits of 1.368(2) Å to 1.388(2) Å. The bond angles of the Cg1 lie between 117.81(15)° and 122.07(15)° (average bond angle = 120°).

The geometries of I and II were comparable with the reported crystal structures (Table S1).^45^ The bond angles of I and II between the pyrazole ring and substituent were compared to the reported compounds 4-(2-(4-chlorophenyl)hydrazinyl)-5-methyl-2-tosyl-1*H*-pyrazol-3(2*H*)-one (PYRA1) and 4-(2-(2,4-difluorophenyl)hydrazinyl)-5-methyl-2-tosyl-1*H*-pyrazol-3(2*H*)-one (PYRA2) and it was found that there is no significant difference in the bond angles except for substitution at the second position of the pyrazole ring. The bond angle between the pyrazole and its second position substituent is around the 117–120° range (in I and II) while the bond angle in this position for PYRA1 and PYRA2 is 128°.

#### 3.1.1 Crystal Packing Features of I and II

Crystal structure is stabilized through the weak C–H···O intermolecular hydrogen bonds in both I and II (Figures S1 and S2 and Table 2: hydrogen bonding is represented by blue dotted lines). Intermolecular hydrogen bonds C8–H8•••O2B (I) and C13–H13•••O2 (II) connect the molecules in the crystal structure. This intermolecular interaction generates *C*(9) chains in I and *C*(8) chain motifs in II.^55^

In II, an additional weak intermolecular interaction (C---H···π) (Figure S2 and Table 2) is observed, which is not observed in I. Weak intermolecular interactions C17–H17···*Cg*2 [C17···*Cg*2 = 3.510 (17) Å, H17···*Cg*2 = 2.66(15) Å, angle = 153(1)°, symmetry 2-*x*,-*y*, 1-*z*] and C18–H18A···*Cg*1 [C18···*Cg*1 = 3.658 (2) Å, H18A···*Cg*1 = 2.96(15) Å, angle = 130(1)°, symmetry −1+-*x*, *y*, *z*] were observed.

### 3.2 Hirshfeld Surfaces Analysis

The quantified intermolecular contacts (Figures S3 and S4) were presented and are as follows: In I, the O···O (1.0%), the O···C(0.4%), the O···H (11.3%), the N···H (8.4%), the C···H (28.1%), and the H···H (50.7%) are the observed intermolecular contacts. The major intermolecular contacts contributing more to the Hirshfeld surfaces were O···H (11.3%), C···H (28.1%), and H···H (50.7%). Similarly, in II: O···H (16.1%), N···C (0.4%), N···H (6.9%), C···C (1.0%), C···H (26.1%), and H···H (49.5%) were the observed intermolecular contacts. The major intermolecular contacts contributing more to the Hirshfeld surfaces were O···H (16.1%), C···H (26.1%), and H···H (49.5%).

In both I and II, the O···H, C···H, and H···H are the common major intercontacts contributing more to the Hirshfeld surfaces. The substitutional effect of methyl groups in the second and fourth positions is clearly visible from the 2D fingerprint plot (Figure S4). The C···H intermolecular contact plot generates a characteristic wing-like projection (arrows in Figure S4) in I, while this is absent in II, showing the possible change in crystal packing of I and II due to the substitutional effect.

### 3.3 DFT

**Figure 3:**
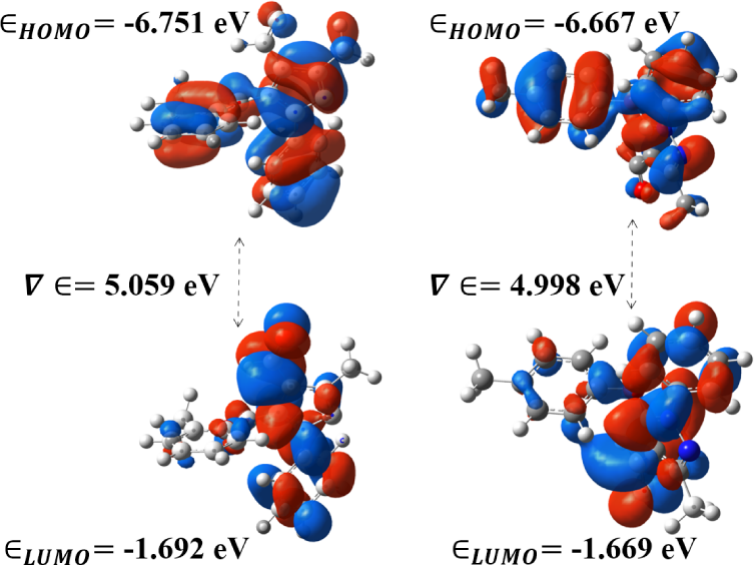
HOMO and LUMO maps of I and **II** compounds.

The visual representation in Figure 3 showcases the 3D plots of the highest occupied molecular orbital (HOMO) and the lowest unoccupied molecular orbital (LUMO) for both pyrazole derivatives. The HOMO corresponds to the highest energy level within a molecule that is currently occupied by electrons. Its significance lies in its role in chemical reactions, as it determines the molecule’s capacity to donate electrons. The HOMO is commonly associated with electron-donating or nucleophilic behavior. Conversely, the LUMO represents the lowest energy level within a molecule that remains unoccupied by electrons. It signifies the orbital that has the potential to accept electrons during a chemical reaction. The LUMO is linked to electron-accepting or electrophilic behavior. The FMO plot of pyrazole compounds demonstrates that the HOMO is distributed widely across the phenyl ring, methylbenzene, and 5-membered ring, indicating its role as an electron donor. In contrast, the LUMO is only spread out on the phenyl ring and 5-membered pyrazole ring, suggesting their inclination toward charge acceptance. All the global reactive descriptors are presented in Table 3, where the energy gap (Δ*E*) is determined to be 5.059 (I) and 4.998 (II) eV, indicating increased stability of the molecule due to a significant energy barrier separating the occupied and unoccupied states. A negative ionization potential signifies a lower energy barrier for electron extraction, making it more likely for the molecule to donate electrons and participate in redox reactions or electron transfer processes.

**Table 3:** Calculated Global Reactivity Properties of the Molecule

	DFT Energy (eV)	
Global Reactivity Descriptors	Mol-1	Mol-2
Band Gap	5.059	4.998
HOMO Energy	–6.751	–6.667
LUMO Energy	–1.692	–1.669
Ionization Potential I= −E_HOMO_	6.751	6.667
Electron Affinity A= −E_LUMO_	1.692	1.669
Global Hardness η= (I – A)/2	2.529	2.449
Electronegativity χ= (I + A)/2	4.222	4.168
Electrophilicity ω=μ^2^/2η, μ= −χ	3.523	3.475

The electrostatic potential (ESP) surface serves as a valuable tool for comprehending the chemical reactivity of a molecule (Figure S5). By examining the ESP study, one can observe that the positive regions indicate H-donor properties, while the negative regions signify H-acceptor properties of molecules. The ESP map illustrates the distribution of the negative potential surrounding the keto oxygen and nitrogen atoms in the pyrazole derivatives. Interestingly, alteration of the position of the methyl group does not affect the negative potential in the system.

### 3.4 Molecular Docking and Molecular Dynamics

**Figure 4:**
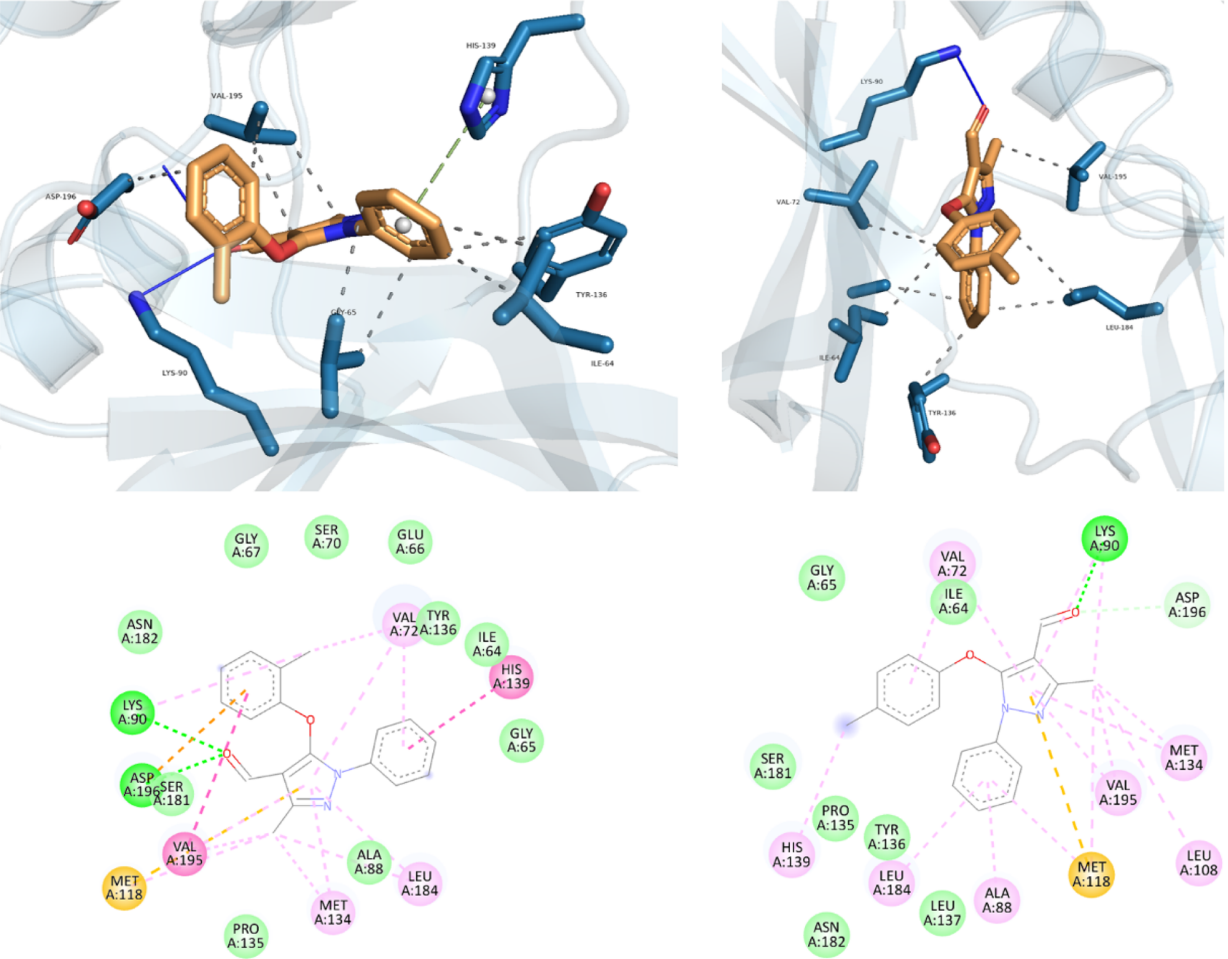
Intermolecular interactions obtained from docking analysis shown in 3D and planar view.

The compounds exhibited strong interactions with CDC7-kinase during molecular docking, with docking scores of −8.459 and −7.784 kcal/mol, respectively. Binding orientations, interactions, and overall conformations of both compounds (I and II) are similar, and thus the binding modes can be considered the same. This indicates a relatively high level of interaction, which is higher than previous reports.^56^ The pyrazole moiety of the compound was found in the binding cavity of hydrophobic and hydrophilic pockets alongside residues Glu66, Ser181, Lys90, Met134, and Val195. Additionally, the keto group in both compounds formed an H-bond interaction with Lys90. The best docking pose for both complexes underwent MD simulation to gain insights into ligand stability within the active site of CDC7 kinase. By analyzing the RMSD values of both complexes over a 100 ns simulation, the binding stability was evaluated. The convergence of RMSD values around a fixed point after each simulation indicates an equilibration of the simulation. The protein–ligand complex in Figure 4 exhibits an RMSD value that indicates the predicted stabilization of the simulation. Throughout the simulation, it is expected for the RMSD values to vary between 1 and 3 Å.

The analysis of the conformational trajectory over time (Figure S6a) reveals that both molecules have an RMSD of less than 3 Å, indicating convergence. This finding aligns with previous reports.^56,57^ Moreover, both complexes demonstrate minimal structural deviation during the 100 ns MD simulations, with an average RMSD value of approximately 2 Å. Larger fluctuations in RMSD could suggest that the ligand has moved away from the binding site, indicating a significant conformational change. To further investigate the flexible regions of the CDC7 kinase protein, root-mean-square fluctuations (RMSF) (Figure S6b) were utilized to analyze the structural fluctuations as a function of the residue number. Large variations corresponding to the loops and turns are indicated by sharp peaks in Figure S6. Additionally, the strength of changes is particularly noticeable at the C- and N-terminal regions of the enzyme. The RMSF of the C- and N-terminal residues of complex-2 is slightly higher than that of complex-1. It is worth noting that both complexes exhibit larger fluctuations in the residues GLN40 and VAL41 compared to other residues in the CDC7-kinase, which can be attributed to the loop region. On the other hand, the ligand-binding residues show smaller fluctuations, indicating that the structure and compactness of the CDC7-kinase are not significantly altered by ligand interaction. The MD simulation results also demonstrate a decrease in the flexibility of residues in the protein–ligand complexes, suggesting a tighter binding. Furthermore, H-bonds, electrostatic interactions, and hydrophobic interactions play crucial roles in the formation of the protein–ligand complex when studying the binding site of CDC7 kinase. During the MD simulation, both compounds exhibit tight binding with minimal movement compared to the docking interactions. Some interactions obtained from docking are no longer present, while new interactions are formed at the end of the simulation. However, the crucial active site residue Lys90 maintains its hydrogen bond with the ligand in compound 2. In contrast, in complex 1, the ligand’s conformation is slightly shifted away from the binding site, and Lys90 forms a hydrophobic interaction. Additionally, the residues Ala88, Tyr96, and Leu184 also engage in hydrophobic interactions, specifically π–alkyl interactions. Moreover, the MM-GBSA technique was employed to calculate the binding free energies for both synthesized molecules. This approach offers distinct advantages over others, such as being less time-consuming and computationally affordable, making it widely applicable. For each complex, the entire simulation trajectory was utilized to estimate the van der Waals and electrostatic components. The results revealed that both compounds exhibit strong binding free energies (Figure S6c). When the two complexes are compared, complex-2 (−44.53 kcal/mol) demonstrates slightly higher binding energy than complex-1 (−41.50 kcal/mol), and both complexes display a more favorable binding free energy at the end of the MD simulation. The increased contribution of van der Waals and electrostatic interaction energy between the ligand and the active site of CDC7-kinase leads to a more favorable binding free energy for the two molecules. Van der Waals energies significantly impact the overall binding energy between a chemical and the target protein.

### 3.5 QTAIM Analysis

Bader’s theory of Atoms in Molecules is used to calculate the topological properties of electron density and the Laplacian of electron density of the chemical bonds of molecules, in which the (3, −1) type of bond critical point (bcp) search was executed^48^ (Figure S7a–c).

**Figure 5:**
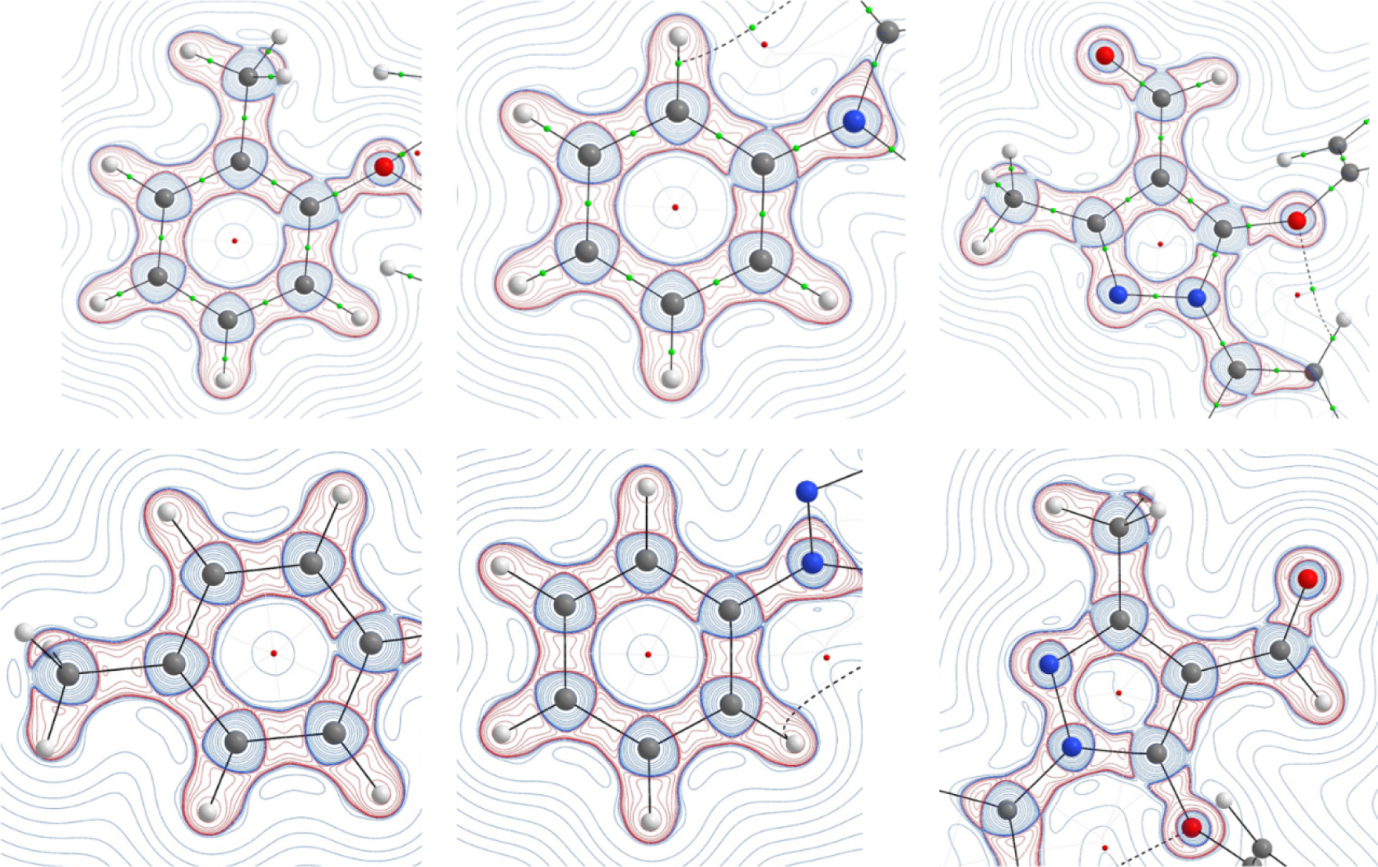
Laplacian of electron density maps of I (top row) and II (bottom row) compounds.

The bonding region, lone pair positions, orientations, charge accumulation area of the atoms in the molecule, and concentration or depletion of the electronic charges of the chemical bonds were identified from the Laplacian electron density (Figure 5).

The topological properties of both pyrazole derivatives were carried (Figure S8a,b). Among all C–C bonds in both molecules, the electron density and Laplacian of electron density of the C12–C13 bond exhibit high values [C7–C8: 2.104/2.115 eÅ^–3^; −20.961/–21.457 eÅ^–5^] due to the bond acting as a double bond character, and the C15–C18 bond carries lower values [C15–C18: 1.688/1.686 eÅ^–3^; −14.175/–14.197 eÅ^–5^] because the bond is present between the phenyl ring and the methyl group.^57^ The high electron density value is noticed in the C(11)=O(2) bond (2.72/2.718 eÅ^–3^); the corresponding Laplacian of electron density is −2.691/-2.72 eÅ^–5^ in both molecules, with the low negative Laplacian indicating that the bonds are highly depleted.^57^

The average C–H bonds of both molecules exhibit a high negative Laplacian, which indicates that the charges of these bonds are highly concentrated. The electron density and Laplacian of electron density of all bonds are presented in Figure 5. This detailed electron density analysis of the polar and nonpolar bonds of the pyrazole molecules is used to investigate the charge concentration/depletion of the chemical bonds.

To gain insights into the inter- and intramolecular interactions present in the crystal structures, we conducted a QTAIM analysis based on the crystal geometries. The findings reveal a single intramolecular interaction observed in both compounds, with the electron density and the Laplacian of electron density for the C8–H8···O2 interaction measured at 0.12/0.13 eÅ^–3^ and 0.37/0.39 eÅ^–5^, respectively. The intermolecular interactions in compound I between O2A and H10C reveal two critical points, exhibiting similar topological properties due to analogous interactions with differing symmetries. The corresponding values are 0.072 eÅ^–3^ and 0.873 eÅ^–5^. In compound II, the O2 atom engages in a similar interaction with C13–H13, which is slightly weaker than that observed in compound I, with values of 0.06 eÅ^–3^ and 0.74 eÅ^–5^. Other C–H···H–C interactions range from 0.046 eÅ^–3^/0.46 eÅ^–5^ to 0.074 eÅ^–3^/0.705 eÅ^–5^, respectively. A comprehensive depiction of weak critical points is illustrated in Figure S13. The NCI isosurface map for all interactions is also provided in Figure S13. The dissociation energy for the intramolecular interaction is calculated to be 9.32 kcal/mol, while the intermolecular interactions exhibit lower dissociation energies, ranging from 2.06 to 4.256 kcal/mol. Notably, all topological parameters align well with previous findings.^58−62^

The intermolecular interactions are a crucial part of rational drug design. Nowadays, the electron densities of these intermolecular interactions are used to determine the ligand function in the active site environment with the help of high-resolution X-ray diffraction. The computational-based charge density study is the method to characterize the intermolecular interaction at the electronic level, due to which the X-ray measurement is yet to challenge the protein–ligand complexes.^57,62^ In this present work, the CDC7 kinase–pyrazole complexes were carried out to extract the topological properties of intermolecular interactions.^54^ The critical point (cp) search on intermolecular interactions gives a (3,-1) type. All the intermolecular interactions of both complexes were stabilized by N–H···O, O–H···O, and C–H···O types of interactions. In complex-1, the electron density ρ_bcp_(*r*) and the Laplacian of electron density ∇^2^ρ_bcp_(*r*) of the N–H/Lys90···O interaction are 0.29 eÅ^–3^ and 3.11 eÅ^–5^, respectively. The ρ_bcp_(*r*) and ∇^2^ρ_bcp_(*r*) of the N–H/Asp196···O interaction are 0.198 eÅ^–3^ and 3.05 eÅ^–5^, respectively. In complex II, the ρ_bcp_(*r*) and ∇^2^ρ_bcp_(*r*) values of the intermolecular interaction between the residue Lys90 and the oxygen atom are 0.36 eÅ^–3^ and 3.32 eÅ^–5^, respectively. The calculated electron density and Laplacian of electron density values of both complexes are in good agreement with reported results.^61,62^ The significant effect of the electron density and Laplacian of electron density is confirmed due to the variation of distance and angle of intermolecular interactions.

Analysis of noncovalent interactions, in combination with Reduced Density Gradient (RDG) analysis, is an essential technique for identifying and characterizing the noncovalent interactions within a molecular system.^61^ This method involves examining the distribution of electron density within molecules to pinpoint areas where attractive and repulsive interactions occur. RDG analysis enables the identification of critical points, such as bond, ring, and cage critical points, which are specific locations in real space electron density where the gradient of reduced density reaches zero. To differentiate between stabilizing and nonstabilizing regions, a chromatic palette is employed, with blue indicating stabilizing interactions and red representing repulsive interactions. The presence of red isosurfaces at the centers of benzene rings emphasizes the impact of ring strain on molecular interactions, particularly strong steric effects. Additionally, the green and brown-colored discs between oxygen/nitrogen and hydrogen atoms signify C–H···O and N–H···O interactions, respectively.

## 4 Conclusions

In this work, 5-(2-methylphenoxy)-3-methyl-1-phenyl-1*H*-pyrazole-4-carbaldehyde (I) and 5-(4-methylphenoxy)-3-methyl-1-phenyl-1*H*-pyrazole-4-carbaldehyde (II) were synthesized. Their three-dimensional molecular structure and crystal structures were determined using a single-crystal X-ray diffraction method. In I and II, the molecules are connected via C–H···O intermolecular hydrogen bonds in C(9) and C(8) ring motifs, respectively. The substitution effect is visualized using 2D fingerprint plots, with a wing-like feature observed in I only, and the type H···H intercontact contributes more toward the Hirshfeld surfaces in both I and II. The docking pose (with hydrogen bonds, intermolecular interactions, and their energy) and protein–ligand complexes (stability in terms of energy over a period) confirm their potential activity against the CDC7 target. The topological properties of I and II revealed the charge concentration and depletion in chemical bonds, similar to the reported compounds. Intermolecular interactions crucial for drug design were characterized using RDG analysis, which identified the critical points and stabilizing/repulsive interactions within the crystal structure.

## Supporting Information Available

Synthesis of intermediate compounds in I and II are presented in experimental section. Packing diagrams of I and II are in Figures S1 and S2. Hirshfeld surfaces (Figure S3), full 2D finger plots (Figure S3) and intermolecular 2D fingerprint plot (Figure S4). Figure S5 shows molecular electrostatic potential map of I and II. Figure S6 shows the RMSD, RMSF, and superimposed map of docked and MD complexes. The bond critical point map, NCI iso-surface map, and RDG scatter of I and II are shown in Figure S7. Figure S8 shows the topological properties of I and II. NMR spectra of I and II are shown in Figures S9, S10, S11, and S12. Bond angles of comparison of I, II, and related compounds are shown in Table S1. The NCI isosurface and critical point maps of I and II compounds are shown in Figure S13 (PDF)
