# Supplementary material for: Experimental, Theoretical, and In Silico Studies of Potential CDC7 Kinase Inhibitors
Source: ACS Omega. 2024 Dec 31;10(1):609–18. doi: 10.1021/acsomega.4c07221 (PMC11740380; doi:10.1021/acsomega.4c07221)
Supplement: Supplementary file 1 — ao4c07221_si_001.pdf [file ao4c07221_si_001.pdf]

An experimental, theoretical and *in-silico* studies of potential CDC7 kinase inhibitors

Kannika Byadarahalli Ravindranath,[1¥] Saravanan Kandasamy,[2¥] Hossam Ebaid,[3] Jameel Al-Tamimi,[3] Sanjeev Murthy,[4] Manju Nagaraja,[5] Ahmad Hosseinizadeh,[6] & Madan Kumar Shankar[7*]

[1]Department of Pharmaceutical Biosciences, Uppsala University, Husargatan 3, Uppsala, 752 37, Sweden
[2]Biological and Chemical Research Center, Faculty of Chemistry, University of Warsaw, Warsaw, 02-089, Poland
[3]Zoology Department, College of Science, King Saud University, Riyadh, 2455, Saudi Arabia.
[4]Department of Chemistry, Sri Siddhartha Academy of Higher education, Tumkur, India
[5]Department of Chemistry, Govt. Degree College, Lingasugur, Karnataka, 584122, India
[6]Department of Physics, University of Wisconsin-Milwaukee, Milwaukee, Wisconsin 53211, USA
[7] Department of Chemistry-BMC Biochemistry, University of Uppsala, Husargatan 3, Uppsala, 75237, Sweden

¥Equal contribution

*Corresponding author: madan.shankar@kemi.uu.se; madan.mx@gmail.com

EXPERIMENTAL SECTION

Synthesis and Crystallization of I

The solution of 2-methylphenol (0.0025 mol) was dissolved in 10 mL of dimethyl sulfoxide, 1-phenyl-5-chloro-3-methyl-1H-pyrazol-4-carbaldehyde (0.002 mol) and potassium hydroxide (0.002 mol) were added and heated on an oil bath at 60 ^{0}C for 6 hours (**Scheme 1**). The final resulting reaction mixture was cooled to room temperature. Later, the reaction mixture was poured into crushed ice, and the separated solid was filtered and washed with water. The dried product was recrystallized from ethanol.

Synthesis and Crystallization of II

The solution of 4-methylphenol (0.0025 mol) was dissolved in 10 mL of dimethyl sulfoxide, 1-phenyl-5-chloro-3-methyl-1H-pyrazol-4-carbaldehyde (0.002 mol) and potassium hydroxide (0.002 mol) were added and heated on an oil bath at 60^{0}C for 6 hours (**Scheme 1**). The final resulting reaction mixture was cooled to room temperature. Later, the reaction mixture was poured into crushed ice and the separated solid was filtered and washed with water. The dried product was recrystallized from ethanol.

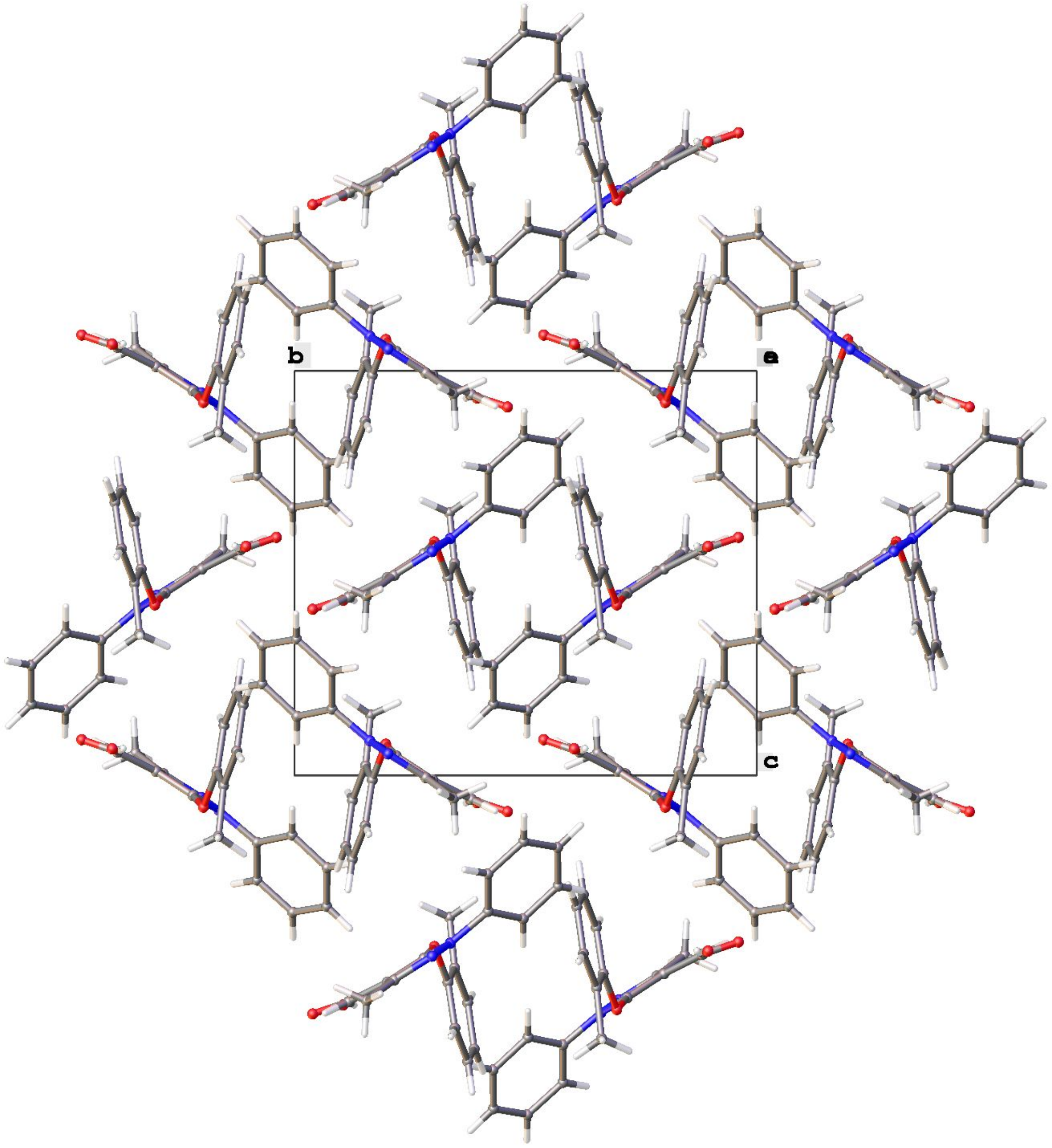

Figure S1. Packing diagram of I along *a*-axis. The *b*- and *c*- axis shown for the reference.

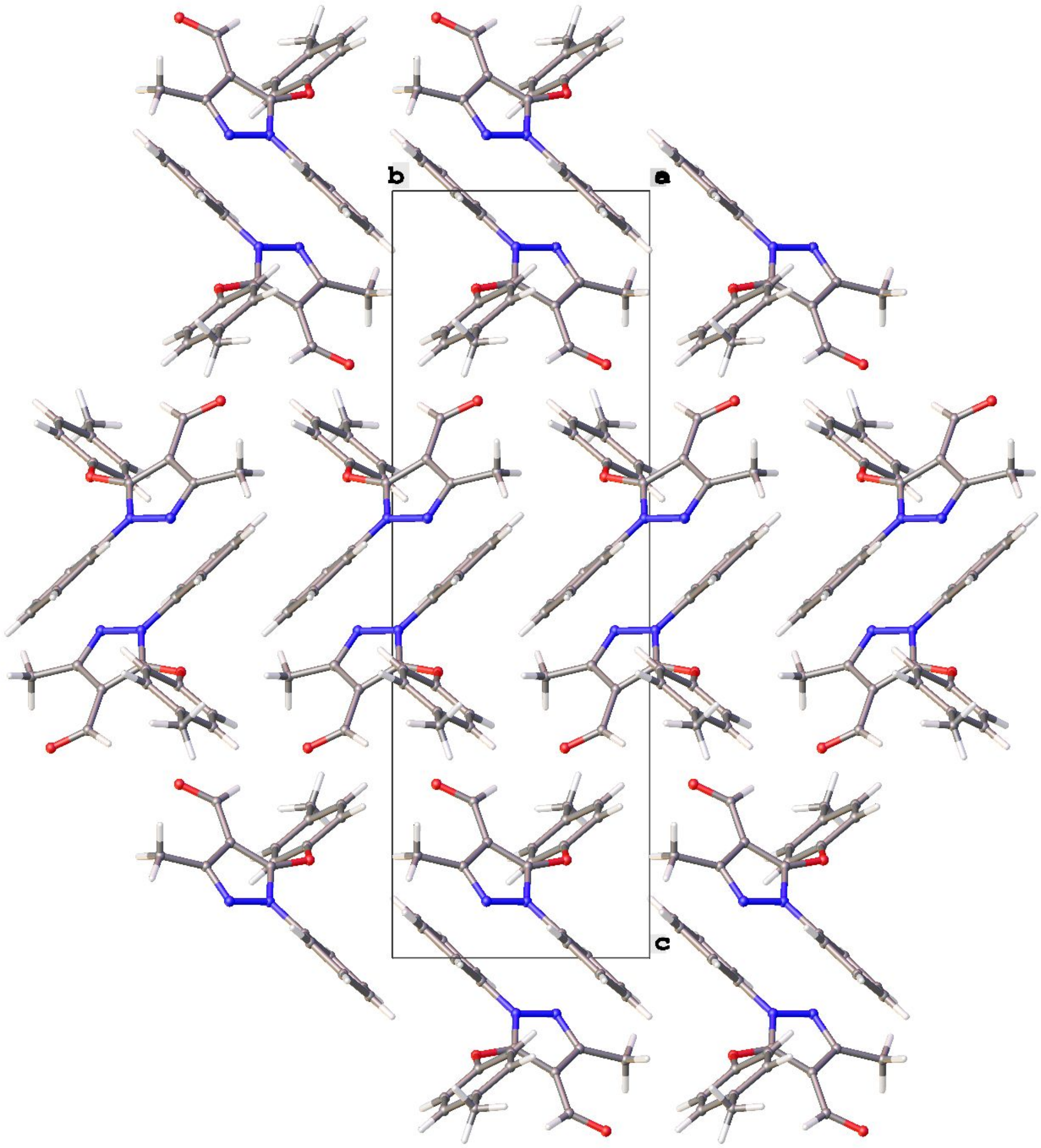

Figure S2. Packing diagram of II along *a*–axis. The *b*- and *c*- axis shown for the reference.

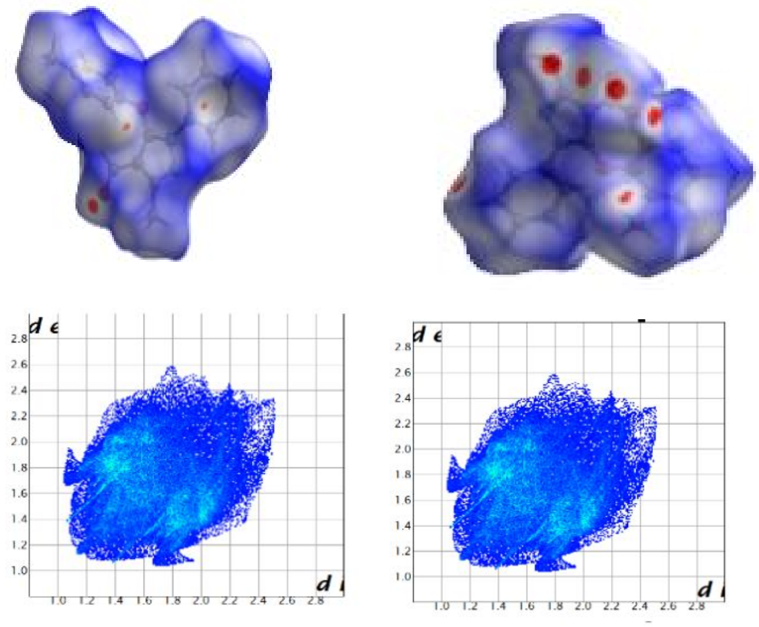

(I) (II)

Figure S3. I and **II;** Top row-Visualization of Hirshfeld surfaces (*dnorm* mapped). Bottom row – 2D fingerprint plot of contributing intermolecular contacts. Color scale -0.030 au (blue) to 1.

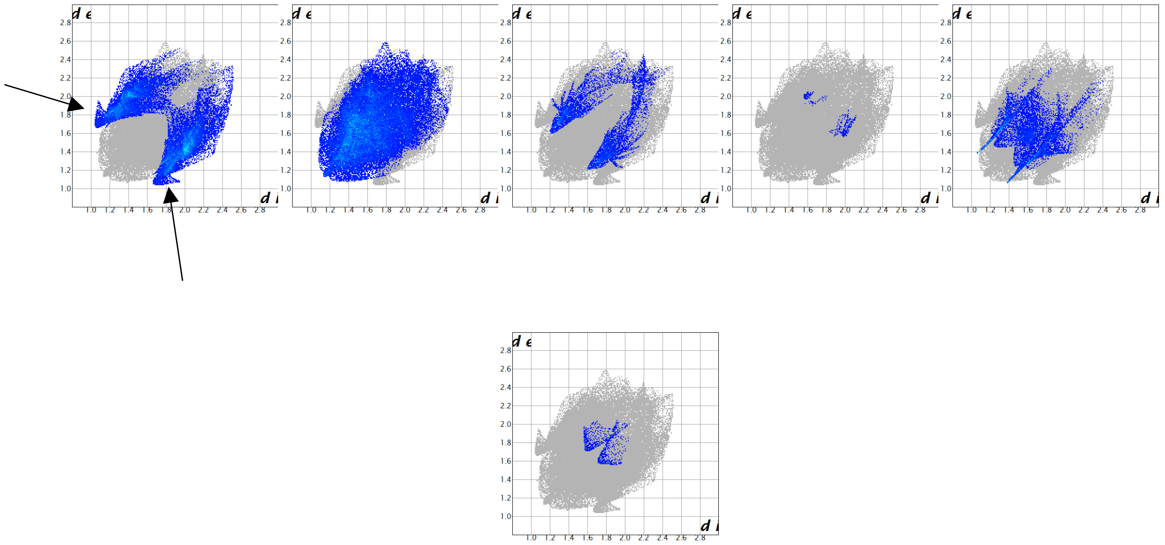

(I)

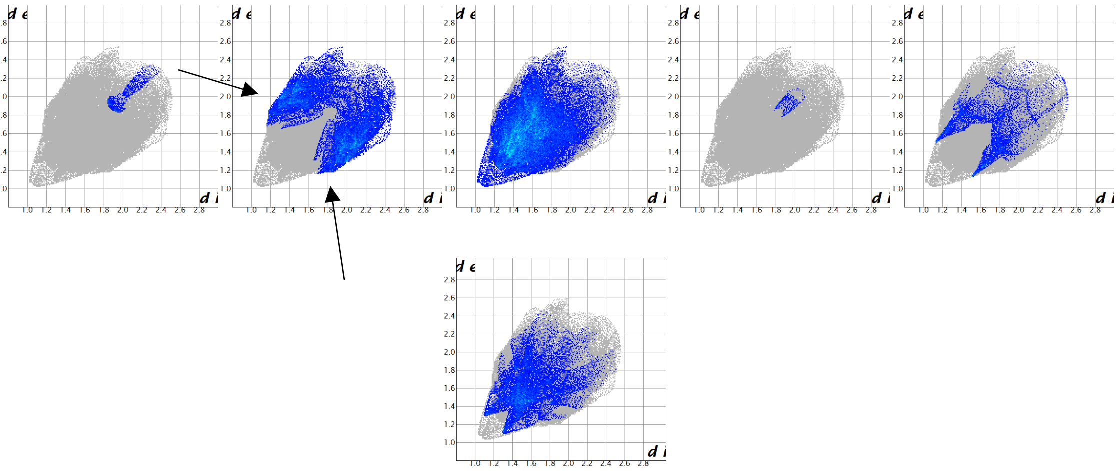

(II)

Figure S4. 2D fingerprint plot of contributing intermolecular contacts in I and II. **(I):** First row: Left to Right: C…H, H…H, N….H, O…C, O…H, Second row: O…O **(II):** First row: Left to Right: C…C, C…H, H….H, N…C, N…H, Second row: O…H

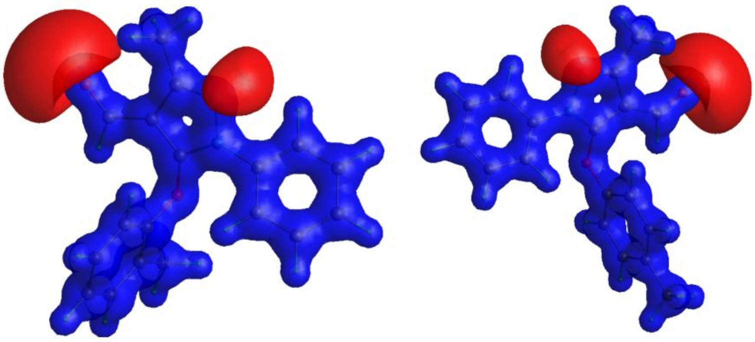

Figure S5. The molecular electrostatic potential map of I (left) and II (right).

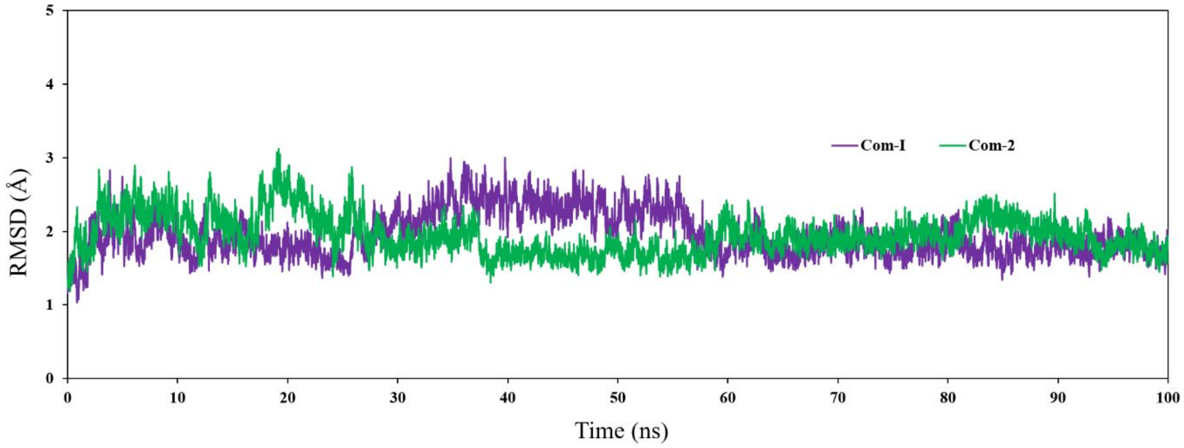

(a)

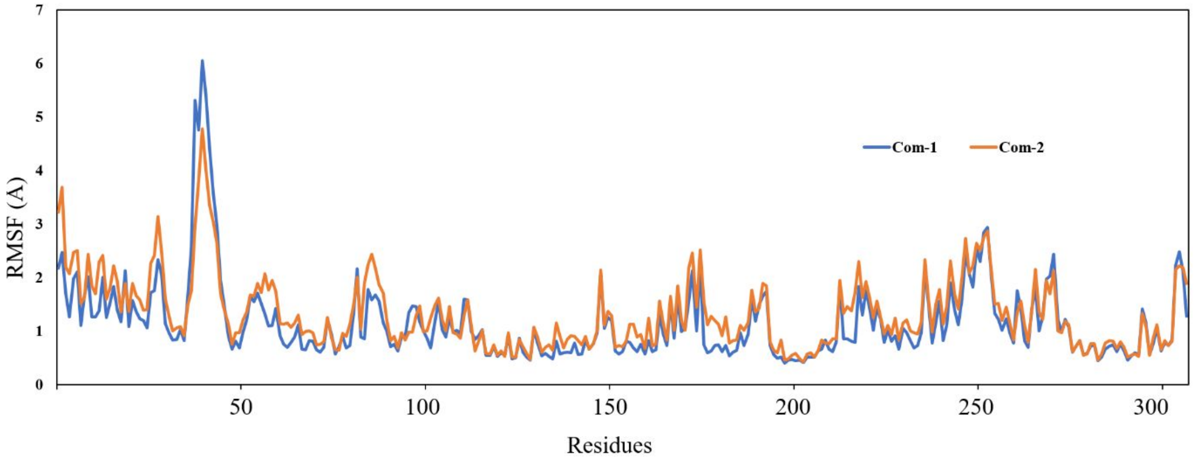

(b)

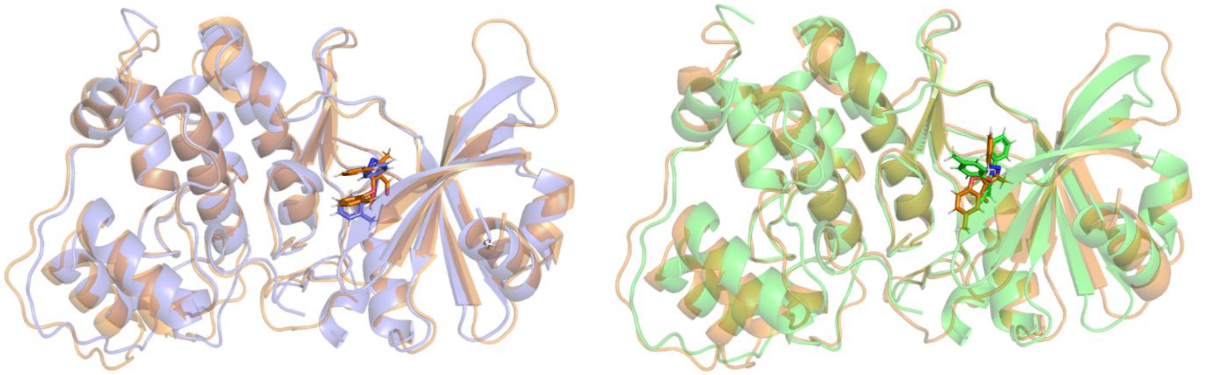

(c)

Figure S6: The (a) RMSD and (b) RMSF plots of both compounds with CDC7 kinase and (c) Superimposed map of both docked and MD complexes.

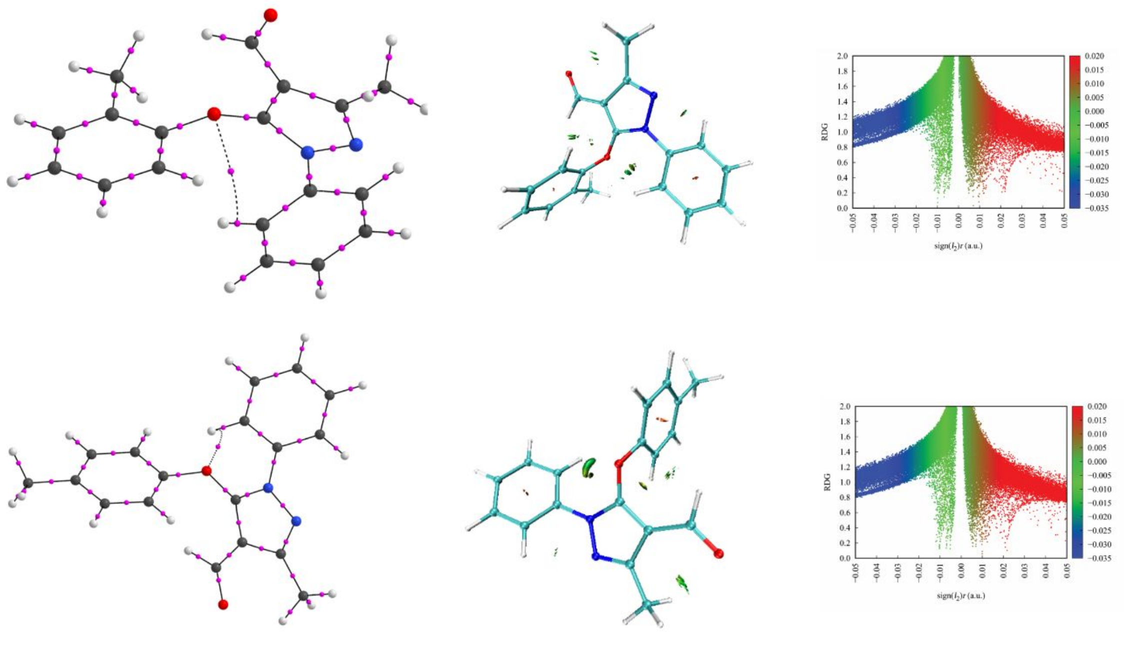

(a) **(b)** **(c)**

Figure S7: The (a) bond critical point (bcp) map; (b) NCI iso-surface map and (c) RDG scatter plots for both compounds.

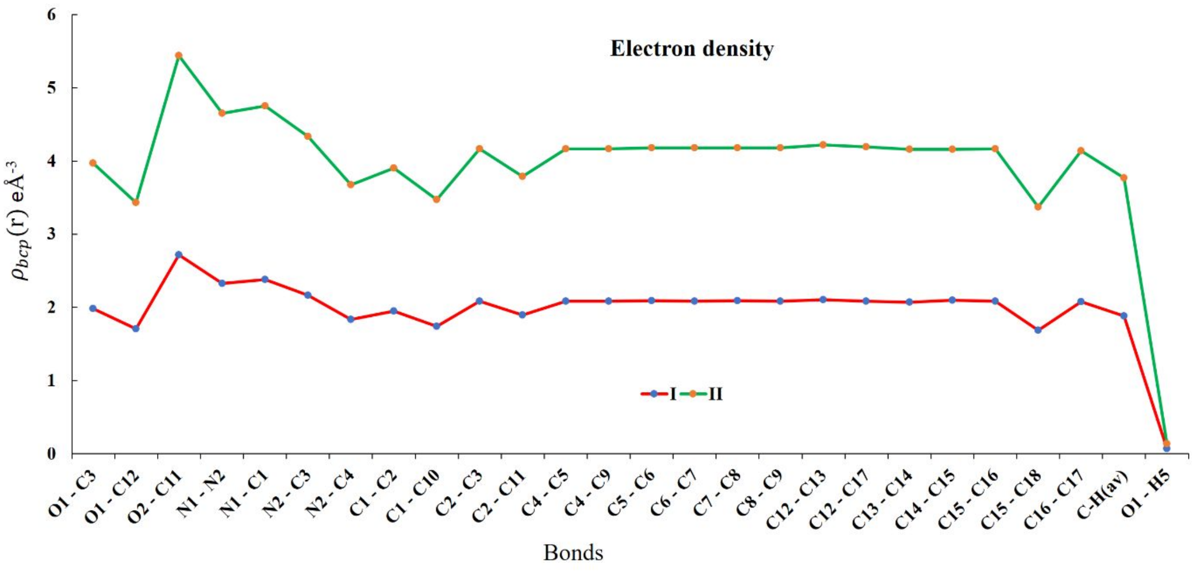

(a)

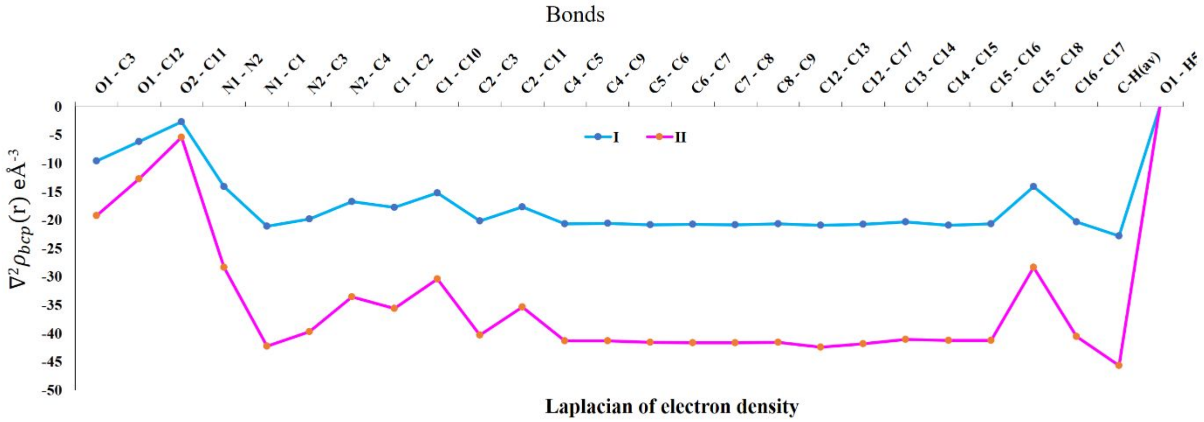

(b)

Figure S8. The compared topological properties (a and b) of I and II.

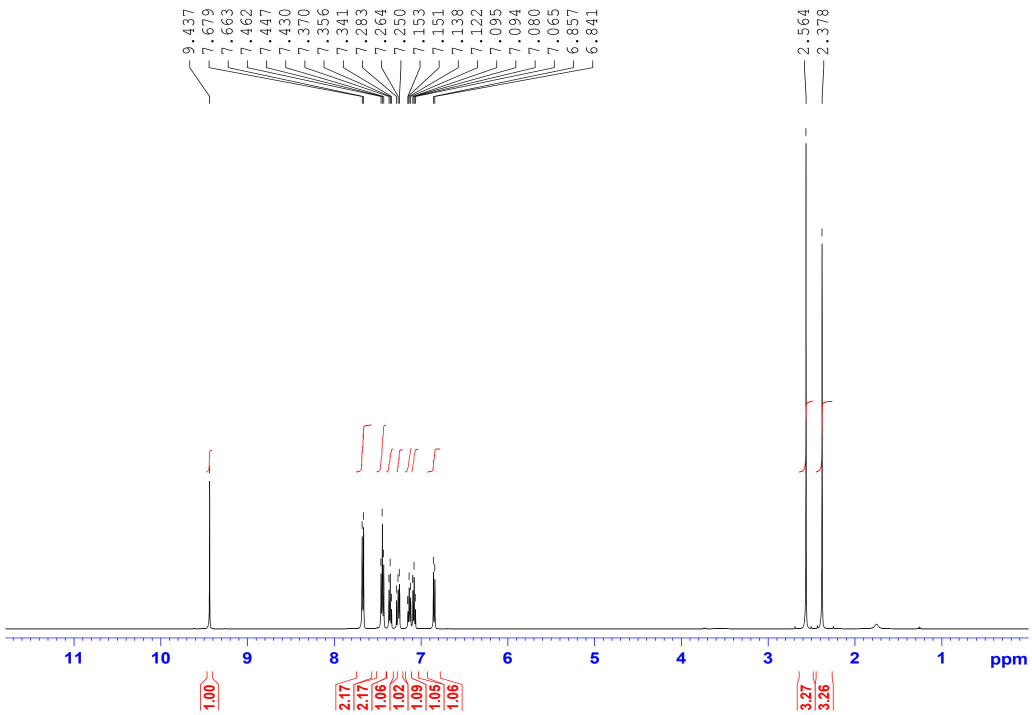

Figure S9: ^{1}H NMR **of** 5-(2-methylphenoxy)-3-methyl-1-phenyl-1H-pyrazole-4-carbaldehyde (**I**)

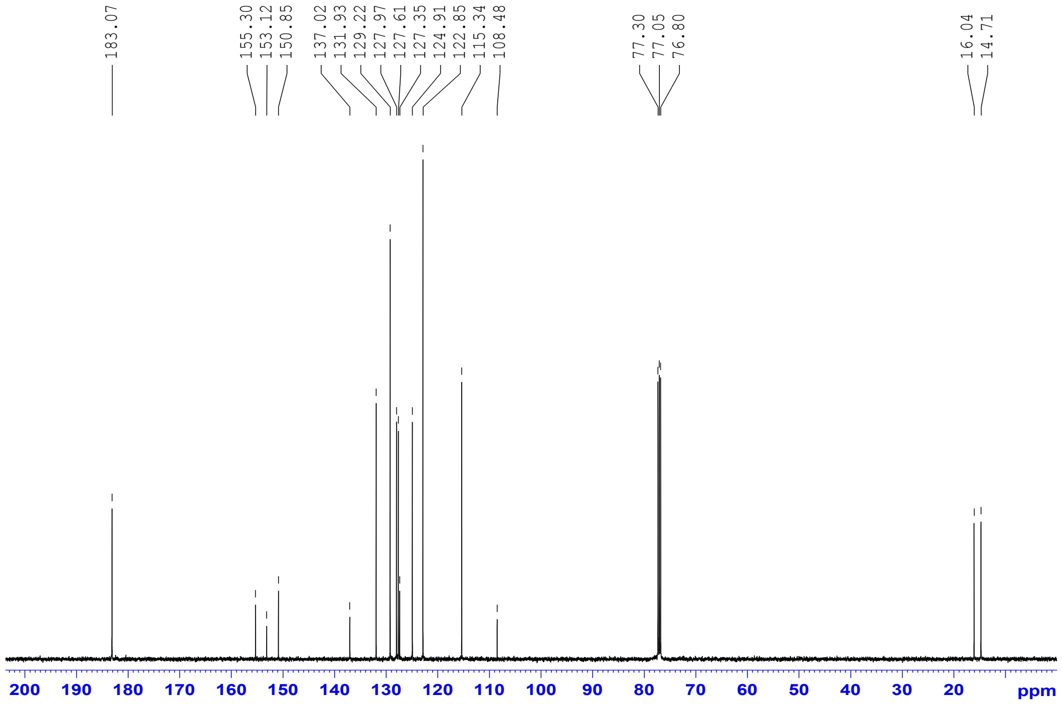

Figure S10: ^{13}C NMR **of** 5-(2-methylphenoxy)-3-methyl-1-phenyl-1H-pyrazole-4-carbaldehyde (**I**)

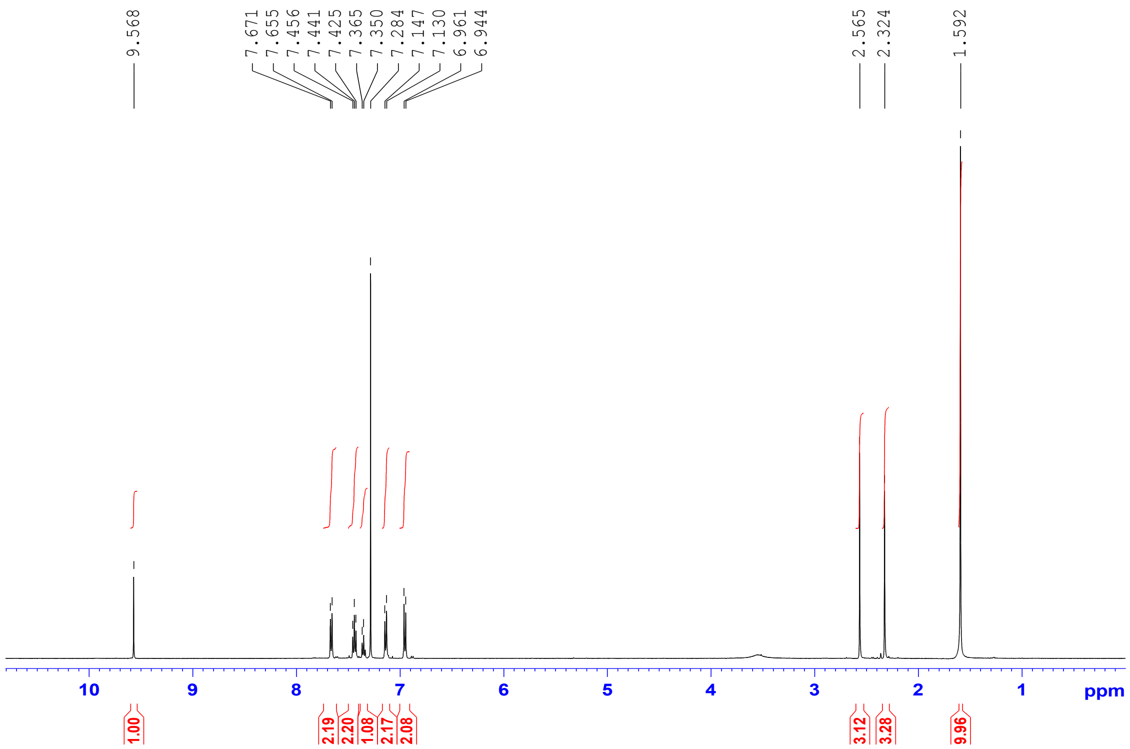

Figure S11: ^{1}H NMR **of** 5-(4-methylphenoxy)-3-methyl-1-phenyl-1H-pyrazole-4-carbaldehyde (**II**)

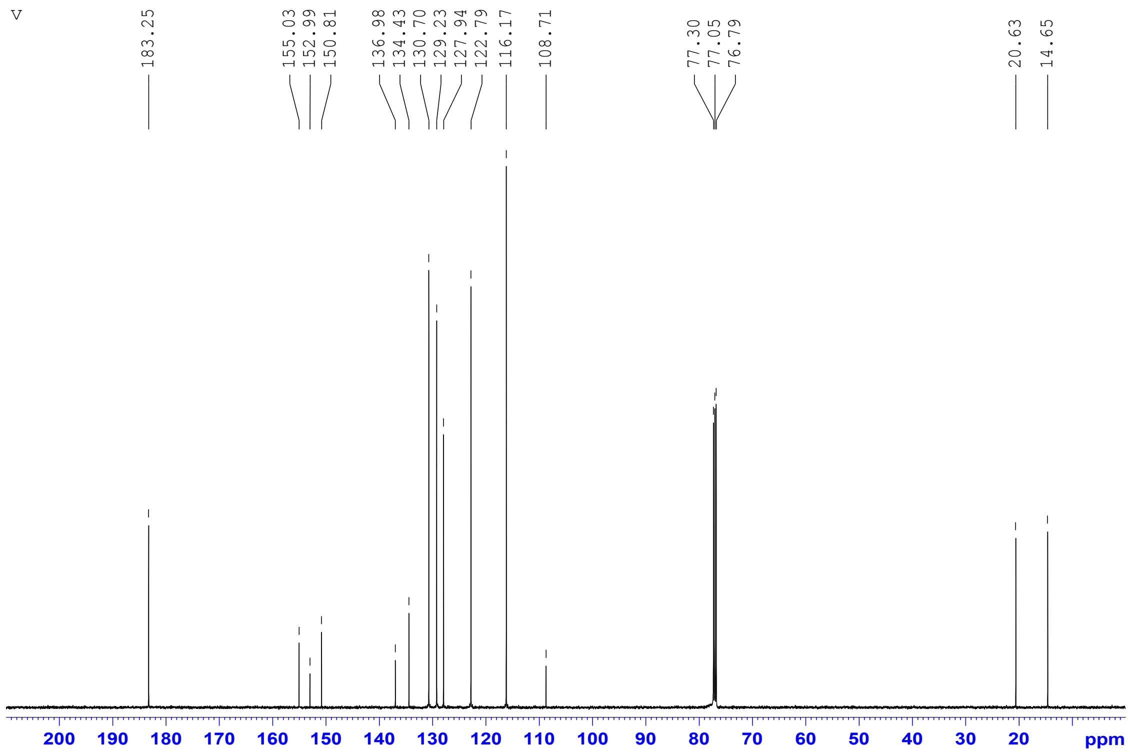

Figure S12: ^{13}C NMR **of** 5-(4-methylphenoxy)-3-methyl-1-phenyl-1H-pyrazole-4-carbaldehyde (**II**).

Table S1: Bond angles of I, II, PYRA1 and PYRA2.

Atoms	I	II	PYRA1 A	PYRA1 B	PYRA2
N1-N2-C4/ N4-N3-S1/	119.5(2)°	119.25(11) °	116.8(6) °	116.4(6) °	117.17(16) °
C3-N2-C4/ C9-N3-S1/ C8-N3-S1	128.7(2) °	129.72(12) °	127.8(6) °	126.9(7) °	128.93(12) °
N2-C3-O1/ N3-C9-O1/ N3-C8-O1	120.1(2) °	117.74(13) °	128.2(8) °	128.4(8) °	128.27(18) °
C2-C3-O1/ C8-C9-O1/ C8-C7-O1	131.7(3) °	133.81(13) °	129.3(8) °	129.3(8) °	129.37(14) °
C2-C3-C11/ C9-C8-N2/ C8-C7-N2	127.9(3) °	128.49(15) °	126.8(8) °	127.2(8) °	127.45(16) °
C1-C2-C11/ C9-C7-N2	127.5(3) °	127.56(15) °	126.3(8) °	125.1(8) °	125.67(17) °

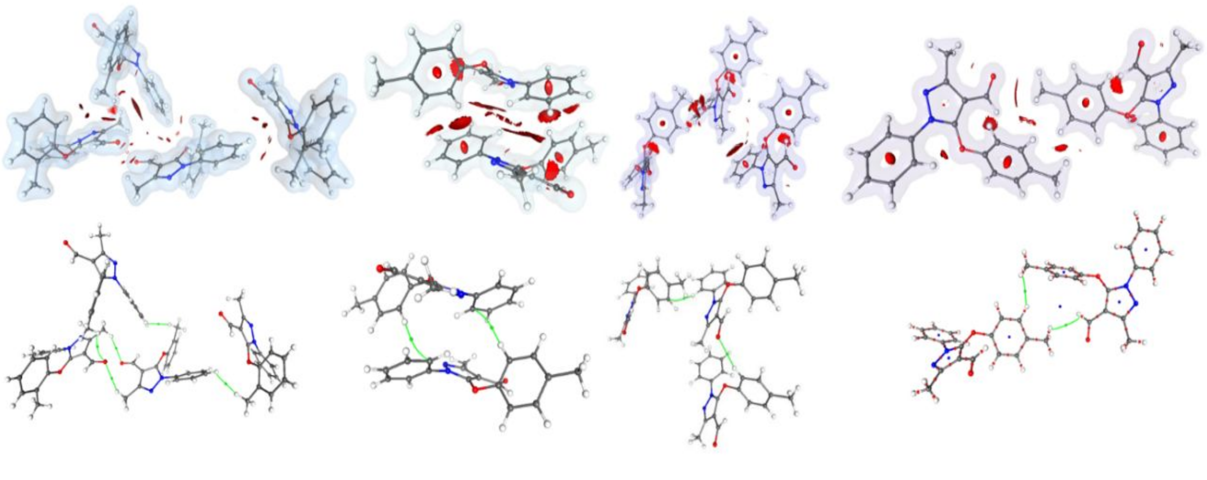

Figure S13: The NCI isosurface and critical point maps of I and II compounds.
